# Crystal structures of highly simplified BPTIs provide insights into hydration-driven increase of unfolding enthalpy

**DOI:** 10.1038/srep41205

**Published:** 2017-03-07

**Authors:** Mohammad Monirul Islam, Masafumi Yohda, Shun-ichi Kidokoro, Yutaka Kuroda

**Affiliations:** 1 Department of Biotechnology and Life Science, Tokyo University of Agriculture and Technology, 2-24-16 Nakamachi, Koganei-shi, Tokyo 184-8588, Japan; 2 Department of Biochemistry and Molecular Biology, University of Chittagong, Chittagong-4331, Bangladesh; 3 Department of Bioengineering, Nagaoka University of Technology, Kamitomioka-cho, Nagaoka, Niigata 940-2188, Japan

## Abstract

We report a thermodynamic and structural analysis of six extensively simplified bovine pancreatic trypsin inhibitor (BPTI) variants containing 19–24 alanines out of 58 residues. Differential scanning calorimetry indicated a two-state thermal unfolding, typical of a native protein with densely packed interior. Surprisingly, increasing the number of alanines induced enthalpy stabilization, which was however over-compensated by entropy destabilization. X-ray crystallography indicated that the alanine substitutions caused the recruitment of novel water molecules facilitating the formation of protein–water hydrogen bonds and improving the hydration shells around the alanine’s methyl groups, both of which presumably contributed to enthalpy stabilization. There was a strong correlation between the number of water molecules and the thermodynamic parameters. Overall, our results demonstrate that, in contrast to our initial expectation, a protein sequence in which over 40% of the residues are alanines can retain a densely packed structure and undergo thermal denaturation with a large enthalpy change, mainly contributed by hydration.

Sequences encoding natural proteins constitute a tiny fraction of the enormous variety of sequences that can be derived from the combination of 20 amino acids. This variety is a major barrier to the elucidation of how a protein structure is encoded in its sequence^1^. However, artificial sequences encoding functional and stably folded proteins have been designed from a reduced set of amino acids^2^,^3^ or by specifying a reduced number of sites along the amino acid sequence^4^,^5^,^6^. This suggests that the information redundancy in a natural protein sequence can be experimentally minimized without compromising its native structure. For example, a chorismate mutase variant encoded with nine types of amino acids^2^ and a bovine pancreatic trypsin inhibitor (BPTI) variant in which 47% of the residues are alanines retain their native functional structures^4^,^5^. Simplified protein sequences that retain their native-like properties are thus expected to allow us to explore the determinants of the thermodynamic stability of globular proteins.

Protein stability remains difficult to rationalize, as most examples of protein stabilization/destabilization result from multiple mutations whose effects are intertwined. A well-documented factor determining protein stability is entropy-driven stabilization^7^, achieved by restricting the conformational freedom of the polypeptide chain in the denatured state by inserting disulfide bonds into it^8^,^9^. However, entropy stabilization can also originate from increased conformational freedom in the native state or even be overturned by enthalpy loss^10^. Enthalpy-driven stabilization may be easier to analyze and control when a highly accurate protein structure, determined at atomic level, is available^11^,^12^. Thus, in practice, the stabilization of protein structure is often achieved through a mixture of rational design and semi-random mutational analysis as the effects of even a modest backbone displacement on protein stability are difficult to quantify.

Here, we report a structural and thermodynamic analysis of six extensively simplified BPTI variants, where 19–24 of its 58 residues are alanines, using differential scanning calorimetry (DSC) and X-ray crystallography. We expected that the replacement of long or bulky side chains with the small alanine methyl group side chains would create destabilizing cavities. However, the alanine substitutions exerted a small unexpected enthalpy stabilization, and the magnitude of which increased in a nearly additive fashion along with the number of alanine substitutions, which was, however, over-compensated with entropy destabilization. A structural analysis indicated that new water molecules were recruited into the spaces created by the alanine substitutions, facilitating protein–water hydrogen-bonded interactions as well as interactions with the methyl group and creating hydration networks around the substitution sites. This observation suggested that enthalpy stabilization originates from hydration rather than from chain enthalpy. This is the very first molecular perspective on hydration enthalpy/entropy, and it suggests a generic strategy for a water-mediated enthalpy stabilization of a protein.

## Results and Discussion

### Design and thermodynamic stability of BPTI variants

**Figure 1:**
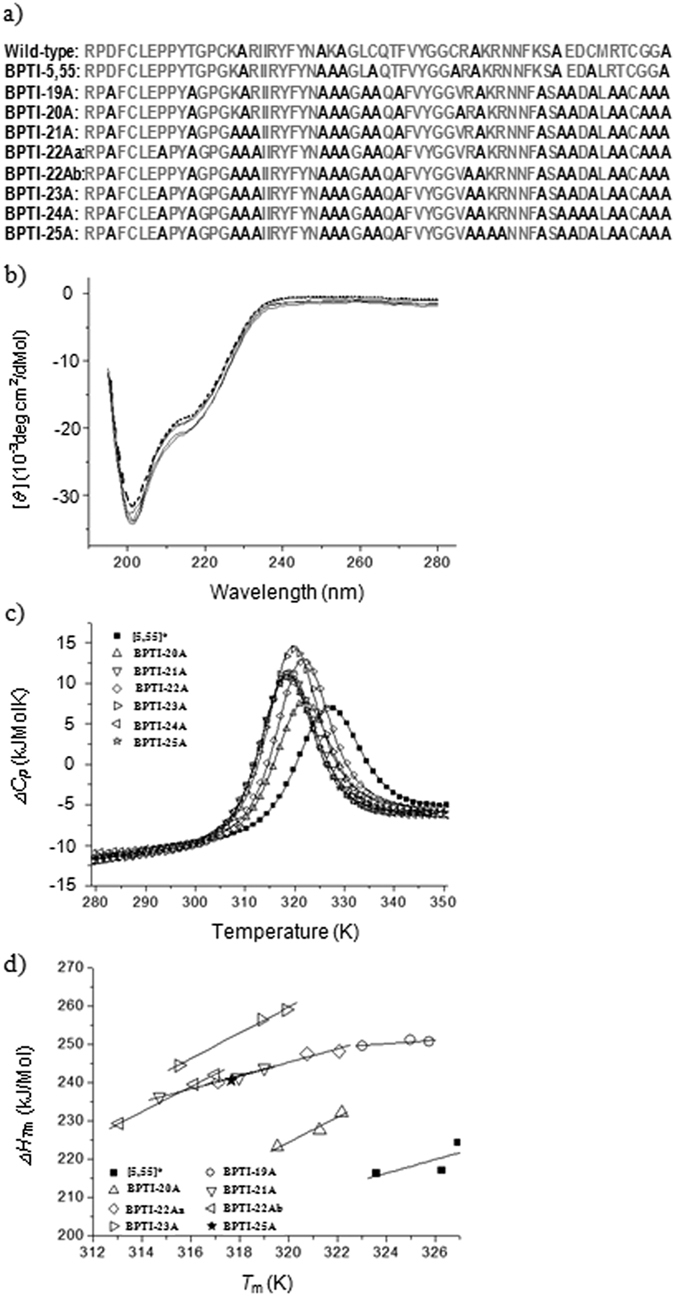
Thermodynamic properties of the simplified BPTI variants. (**a**) Sequences of the simplified BPTIs. Alanines are shown in black, and the other residues are in gray. (**b**) CD measurements at 20 μM concentration in 20 mM Acetate buffer (pH4.7) and at 4 °C. The dotted line represents the reference BPTI-[5,55] A14GA38V^12^, and the continuous lines stand for BPTI-19A, -22A and -25A variants. Similar patterns were also observed at 40 °C and 70 °C (Suppl. Figure S1). (**c**) Thermal stability measurements using differential scanning calorimetry at pH4.7. The symbols represent the experimental data and the continuous lines represent two-state model fitted curves. (**d**) Temperature-dependence of the enthalpy change (∆*H*) upon thermal unfolding of BPTI-[5,55] A14GA38V and extensively simplified BPTIs. The pH values are 4.1, 4.7 and 5.5, from the lowest to the highest *T*m values. 5,55* stands for BPTI-[5,55]A14GA38V.

We used a stabilized BPTI-[5,55] (BPTI-[5,55] A14GA38V^12^), containing only the 5–55 disulfide bond, as the reference molecule instead of wild-type BPTI, which contains three disulfide bonds^13^, and a BPTI variant containing 19 alanines (BPTI-19A) as the template for further simplification^14^. We mutated the exposed and partially exposed residues (P8, K15, R17, R39, K41, R42, and D50) to alanines because we expected that these substitutions would minimally affect the densely packed interior of the native protein^4^,^5^. The mutants were named according to the numbers of alanines in their sequences (i.e. BPTI-19A stands for a BPTI variant containing 19 Alanines) and a suffix ‘a’ or ‘b’ was added to distinguish variants containing the same numbers of alanines at different sites^4^,^5^ (Fig. 1a).

Circular dichroism measurements indicated that all the simplified BPTI variants had the same secondary structure contents as the reference BPTI-[5,55] A14GA38V (Fig. 1b). Thermal denaturation curves, measured with DSC, were all cooperative and two-state, typical of a small natively folded protein with a densely packed interior (Fig. 1c,d; Suppl. Figure S1). Substitution with alanine slightly reduced the melting temperature (*T*_m_) of the variants, as anticipated. Interestingly, the ∆*C*_*p*_ values also increased slightly with increasing number of alanines in the sequences (Table 1). However, unexpectedly, the enthalpy changes at both *T*_m_ (∆*H*_*T*m_) and 37 °C (∆*H*_37 °C_) increased as the number of alanine substitutions increased (Table 1 and 2). This increase in enthalpy was counter-intuitive, because we imagined that the substitution of bulky residues with the small alanine side-chain would create voids, thus reducing the van der Waals interactions between side-chain atoms and consequently the enthalpy of denaturation. Finally, the thermodynamic parameters estimated at 37 °C clearly indicated that the enthalpy stabilization was over-compensated by entropy destabilization (Suppl. Figure S2b), slightly reducing the melting temperatures (Suppl. Figure S2a; Table 2; and Suppl. Table S1).

### Crystallization and structures of the simplified BPTIs

**Figure 2:**
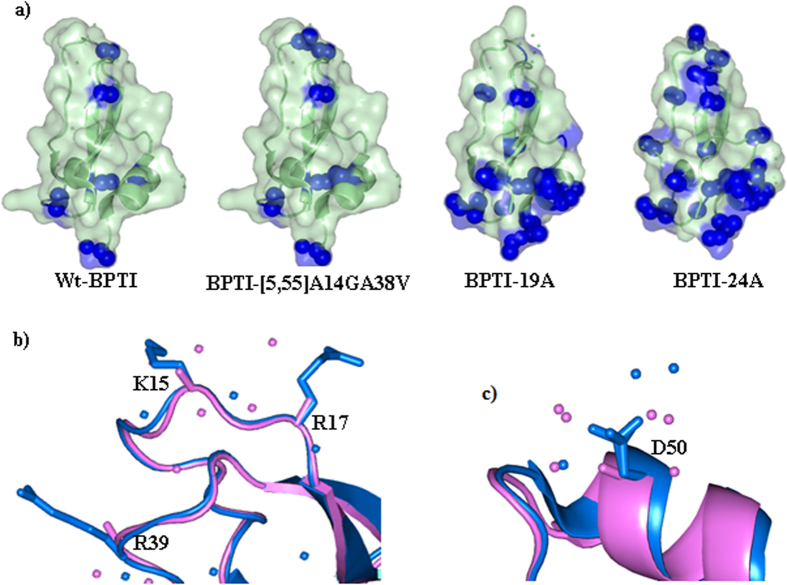
Structures of the simplified BPTI variants. (**a**) Structures of wild-type BPTI, BPTI-[5,55]A14GA38V, BPTI-19A and BPTI-24A, from left to right. Alanines are shown as blue spheres in surface model. New hydration networks around the K15AR17A (**b**) and D50A (**c**) sites. Wild-type BPTI and BPTI-24A are shown with a ribbon model in blue and violet respectively. Spheres represent water molecules around the alanine substitution sites.

**Figure 3:**
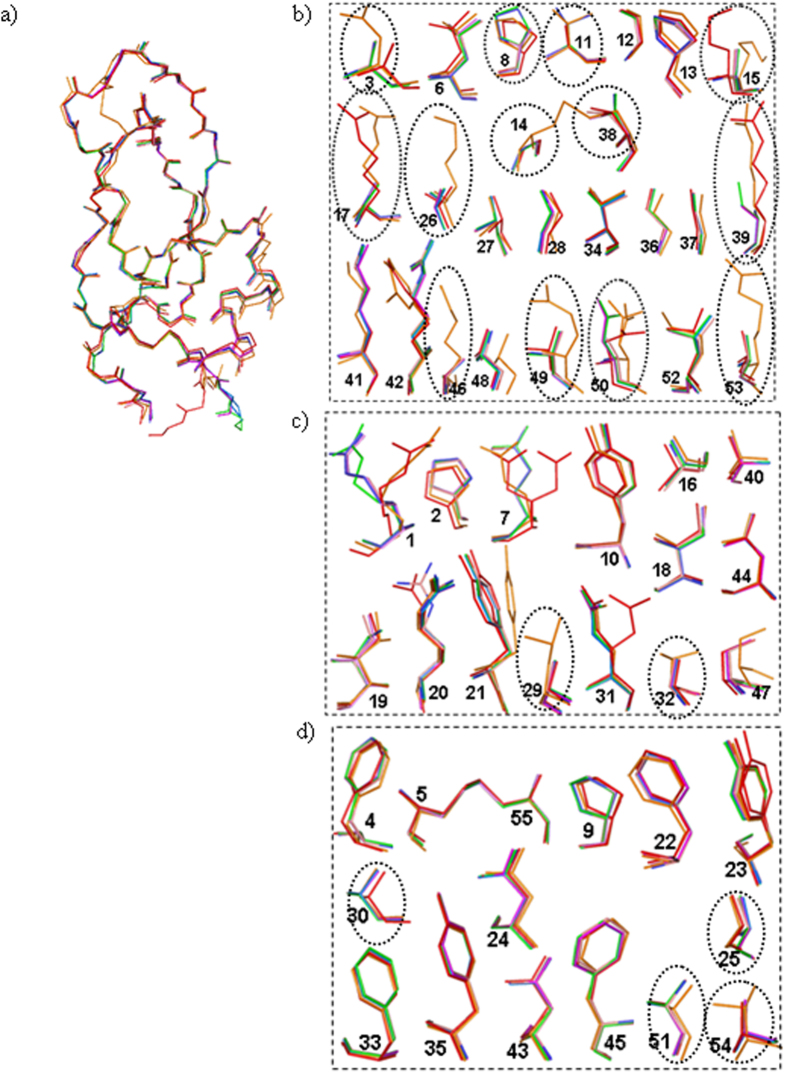
Structural details of the simplified BPTI variants. (**a**) Superimposition of simplified BPTIs onto 2SS-BPTI (7pti.pdb). The overall structures remained almost unchanged with RMSD < 0.4 Å. Side-chain conformation of surface exposed (ASA > 50%), partially buried (ASA 30–50%) and buried (ASA < 30%) residues are shown in panels b, c, and d, respectively. In panels b-d the alanines as well as the residues substituted to alanines are encircled. The side-chain conformations of almost all residues were retained in all simplified BPTIs, indicating that multiple alanine substitutions did not affect the native-like densely packed protein interior. In all panels color codes are the same (7pti: orange, BPTI-19A: red, BPTI-21A: green, BPTI-22Ab: blue, BPTI-23A: yellow, and BPTI-24A: violet).

To provide a structural view of this peculiar instance of enthalpy stabilization, we crystallized and solved the structures of six simplified BPTI variants containing 19, 20, 21, 22, 23, and 24 alanines (Fig. 2; Table 2). On the other hand, despite repeated trials, BPTI variants containing 25–27 alanines merely formed needle crystals, unsuitable for diffraction. All six variants crystallized under the same conditions, indicating a minimal disturbance of the surface properties, even upon multiple alanine substitutions. The alanines were spread almost uniformly over the entire BPTI structure (Fig. 2a). In all of the structures, the overall backbone and side-chain conformations were almost perfectly retained, with a backbone root square mean deviation (RMSD) of 0.3–0.4 Å, which is similar to the RMSD of the wild-type BPTIs solved by different research groups (Table 3; Fig. 3; Suppl. Figure S3). Therefore, despite the large number of alanine substitutions, the native-like backbone structure and the densely packed protein interior remained completely unaffected (Fig. 3 and Suppl. Figure S4).

**Figure 4:**
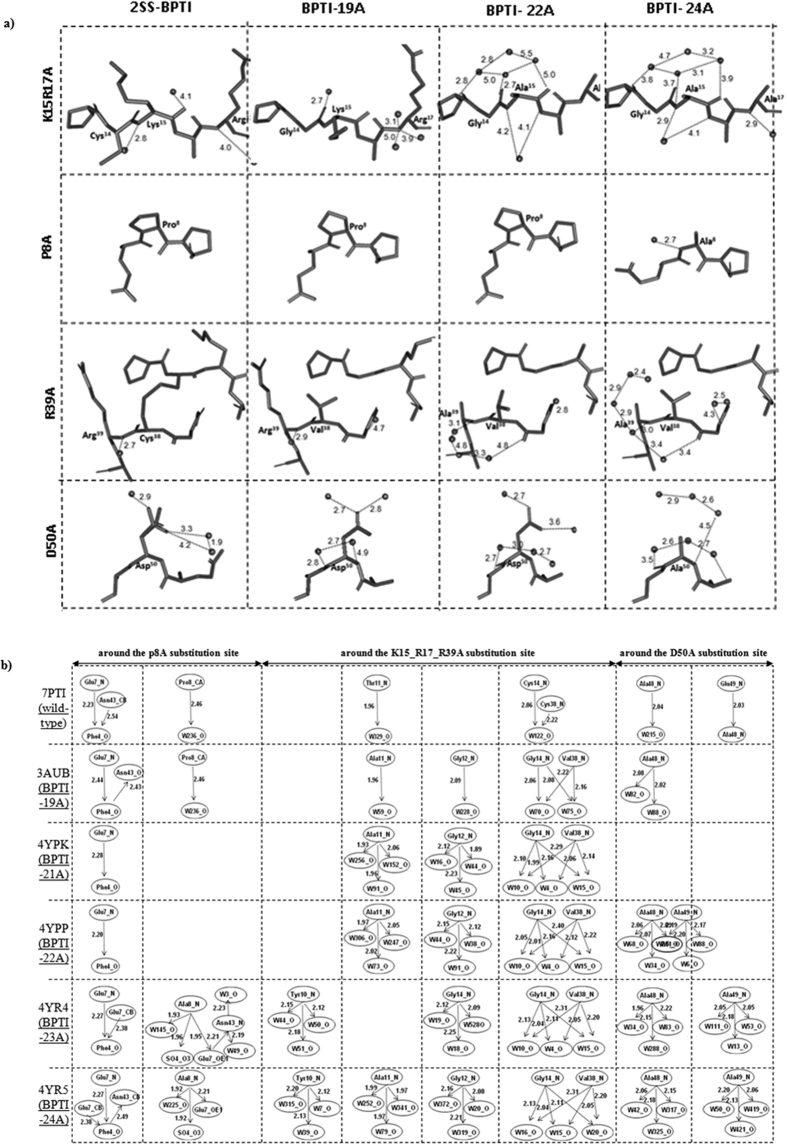
Hydration structures in 2-SS BPTI and simplified BPTIs. (**a**) New hydration networks around the larger side-chains to smaller alanine substitution sites. Alanine substitutions introduced in this study are mentioned on the left of the panel and BPTI variants are indicated at the top of the panel. Chain A of BPTI-19A, BPTI-22Ab and BPTI-24A were globally superimposed onto the structure of 2SS-BPTI (7PTI.pdb) using PyMol (www.pymol.com), and the individual alanine substitution sites are shown at identical scale. Inter-atomic distances between backbone atoms (amide nitrogen and carbonyl oxygen) and water molecules are mentioned in Angstrom. (**b**) New hydrogen bond forming water molecules. Arrows indicate the direction of the hydrogen bonds, from donor to acceptor atoms. The number of protein-water hydrogen bonds increased at and around the substitution sites with increasing number of alanine substitutions. At sites far from the alanine substitutions the hydrogen bonding and hydration structures remained unchanged (Suppl. Figure S4). Protein-water hydrogen bonds were calculated using HBAT (26). Residues identities and inter-atomic distances (Å) are mentioned.

For the purpose of discussion, we examined the fine structural changes induced near the substitution sites by the mutations. The K15A and R17A substitutions, which are located in a loop, did not affect either the local main-chain or the side-chain structures (Figs 2 and 3; Suppl. Figure S4a), but two new water molecules were recruited near the amide nitrogens of Tyr^10^, Ala^11^, Gly^12^, and the Gly^14^/Val^38^ pair to fill the spaces left by the large side chains after the alanine substitutions (Fig. 4). Seven novel water molecules appeared near residues 10–14 and the Gly^14^/Val^38^ pair, forming an elongated hydration network involving water molecules hydrogen-bonded to the backbone atoms of the protein (Fig. 4). The R39A substitution also recruited 1–2 new water molecules close to the amide nitrogen of Ala^39^, extending the hydration networks observed in BPTI-21A. Similarly, the P8A substitution did not affect the local structure, but recruited a water molecule and a sulfate ion, which formed new hydrogen bonds with the amide nitrogen of Ala^8^ (Fig. 4). Ala^8^ was also hydrogen-bonded to the ε Oxygen atom of Glu^7^ (OE1) which was further hydrogen-bonded to Asn^43^ and two new water molecules (Fig. 4a,b). This hydration structure is absent from the wild-type BPTI structures and from all the simplified BPTIs containing Pro at the 8^th^ position (Fig. 4b). The D50A substitution in the α-helix did not affect the backbone conformation around residue 50 (Fig. 2c), but four new water molecules were recruited: two near the amide nitrogen of Ala^48^ and two near the amide nitrogen of Ala^49^ (Fig. 4). To date, only a single water molecule is observed at these sites in the wild-type and 2SS-BPTI structures, whereas in the simplified structures, three water molecules were hydrogen-bonded to Ala^48^ and Ala^49^ (Figs 2c and 4). Finally, both the intramolecular hydrogen bonds and the hydration structures at other positions were essentially unchanged from those in the wild-type BPTIs (Suppl. Figure S4). These observations clearly indicate that the multiple alanine substitutions merely affected the native-like structure of BPTI, but new water molecules appeared in the vicinity of the main-chain atoms near the substitution sites (Figs 2b,c and 4).

### Structural interpretation of the thermodynamic parameters

**Figure 5:**
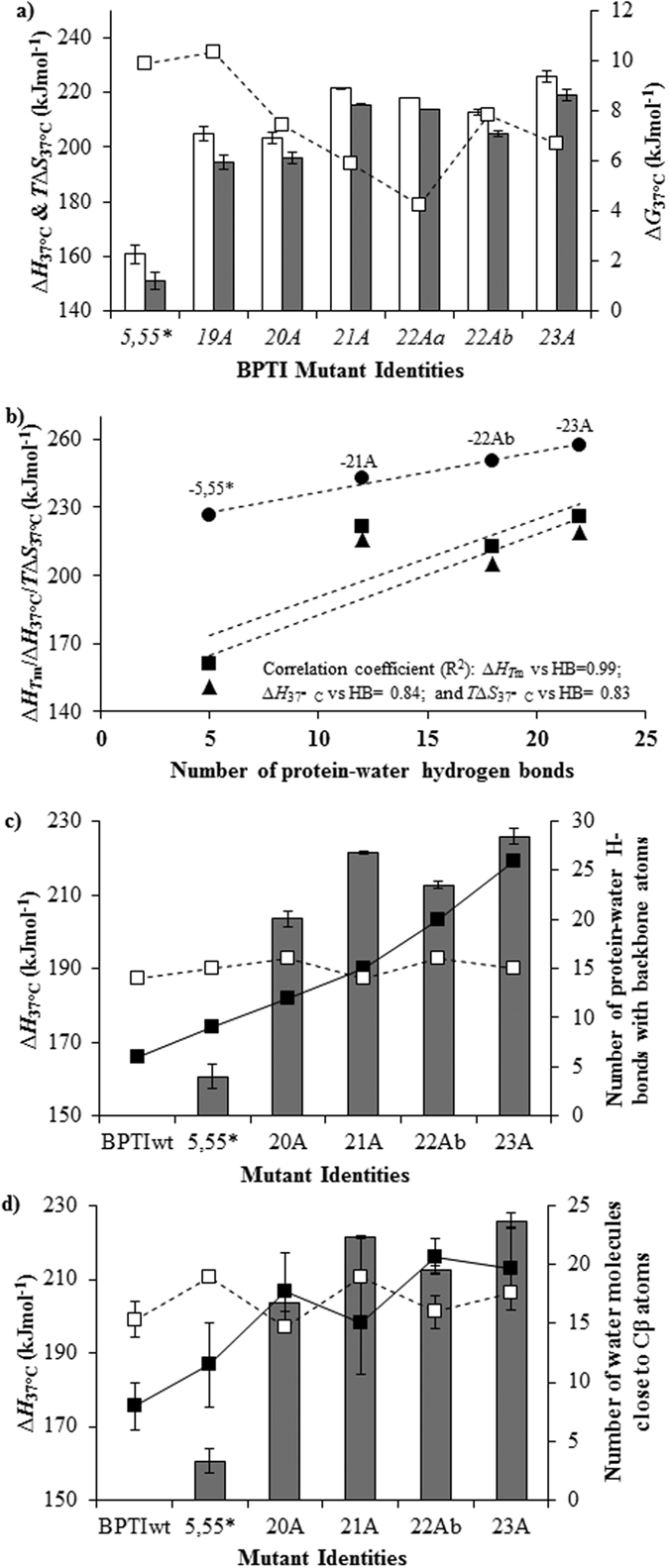
Correlation between thermodynamic parameters and hydration networks around the alanine substitution sites. (**a**) Specific thermodynamic parameters estimated at 37 °C are shown along the horizontal axis (□ open bars: changes in enthalpy (*∆H*_37 °C_ kJ/mol) and 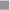 gray bars: changes in entropy (*T∆S*_37 °C_ kJ/deg.mol)) are shown and along the vertical axis (□ open squares represent the changes in free energy (*∆G*_37 °C_ kJ/mol)). (**b**) Correlation plot of thermodynamic parameters [●:enthalpy change at *T*_m_ (*∆H*_*T*m_); ■: enthalpy change at 37 °C (*∆H*_37 °C_); and ▲: entropy change at 37 °C (*T∆S*_37 °C_)] versus the protein-water hydrogen bonds observed around the alanine substitution sites in their crystal structures (see also Materials and methods; and Fig. 4 legends). Correlation coefficients are shown within the panel. *∆H*_37 °C_ kJ/mol versus the number of protein-water hydrogen bonds to the backbone atoms and versus the number of water molecules close to Cβ-atoms are shown in panels c and d, respectively. Water molecules residing within 3.4 to 4.0 Å from side-chain Cβ-atoms were considered as ‘close’. In panel c, we considered 7pti as wild-type BPTI and 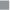 bars, ■ and □ squares represent, respectively, *∆H*_37 °C_ kJ/mol, the number of protein-water H-Bonds around the alanine substitution sites, and those at sites not substituted to alanines. In panel d, we considered 5pti, 6pti and 7pti structures as wild-type BPTI and for the simplified variants we included all the chains (monomers) in their asymmetric unit. The 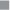 gray bars stand for *∆H*_37 °C_ kJ/mol. The ■ and □ squares represent the number of water molecules close to Cβ atoms of, respectively, alanines and amino acids different from alanines. The number of water-water H-Bonds and number of water molecules close to the Cβ-atoms of alanines increased with increasing alanine substitutions while the numbers remained almost the same at residues not substituted to alanines. Similar correlation was also observed in specific entropy versus hydration structures plot (Suppl. Figure S5). 5,55* stands for BPTI-[5,55]A14GA38V.

Let us consider possible structural features that could account for the increase in enthalpy change arising from the multiple alanine substitutions (Fig. 5). X-ray crystallographic analyses indicated that all of the simplified BPTI structures fully overlapped (Fig. 3) and that the main change that occurred upon the number of alanines was an increase in the solvent content in the asymmetric units (Table 3). The new water molecules were recruited around the alanine substitution sites and were involved in novel hydration networks (Figs 2, 4 and 5). Thus, favorable protein–water interactions appear to be the most likely factor responsible for the increased unfolding enthalpy of the simplified BPTIs, rather than the relaxation of atomic clashes or the creation of new intramolecular hydrogen bonds or van der Waals contacts (Figs 4 and 5), assuming that the hydration structures remain the same for all alanine mutants in their denatured states.

Generally, an increase in entropy change is interpreted as either an enlargement of the conformational space in the denatured state or as a loss of it in the native state, in which both the chain and hydration terms must be accounted for. We first imagined that replacing bulky side chains with small alanine side chains would create voids, increasing the flexibility of the local chain in the native state, and thus reducing the entropy of unfolding, and consequently stabilizing the native state in terms of entropy. Another possibility being that the entropy of the denatured state increases by reducing the size of side-chains and thus increasing the conformational space of the denatured state, which would destabilize the native state. DSC experiments indicated that the entropy of unfolding increased upon replacement of the native amino acids with increasing number of alanine replacements. Moreover, the crystal structures indicated that the voids were filled with water molecules and the flexibility or dynamics of the residues surrounding the mutations were unchanged, as assessed with the B-factors (Suppl. Figure S3). The entropy destabilization observed upon alanine substitution may thus originate from the enlargement of the conformational space in the denatured state. For example, a P → A mutation is estimated to result in a reduction of 3.5 °C in the melting temperature mainly destabilized by entropy^7^ and these figures are roughly consistent with a *T*_m_ reduction of 2.24 °C observed upon the P8A substitution (Table 2). The difference could be accounted for by entropy loss associated with the increased number of water molecules released to the bulk water upon the denaturation of the simplified BPTIs (Fig. 5c,d). Finally, a strong correlation between the number of protein–water hydrogen bonds (Fig. 5b,d), as well as the number of water molecules interacting with the newly added methyl groups^15^ (Fig. 5c), and the thermodynamic parameters further substantiated the notion that these water molecules represent the molecular origin of hydration enthalpy/entropy. In principle, measuring the thermodynamics of mutants where a large buried hydrophobic residue is replaced to alanine could provide further proof of this effect, however, such substitutions nearly completely unfold BPTI-[5,55]^16^ making such analysis impractical.

## Concluding remarks

The enthalpy stabilization introduced to a protein with multiple alanine substitutions is novel and unexpected. A comparison of the thermodynamic parameters and structural data suggests that the enthalpy stabilization of the simplified BPTIs probably arises from improved interactions between water molecules and the protein. This observation sheds new light on the molecular nature of the hydration term of enthalpy/entropy of unfolding. Further analyses, such as DSC performed with D_2_O protein solutions might enable decomposition of water contribution to electrostatic and hydrogen-bonding terms.

The rational design of enthalpy stabilization usually requires high-resolution structures for designing novel hydrogen bonds^17^ or salt bridges^18^ to fill cavities^19^ or to relax steric clashes^11^,^12^, which is difficult. On the other hand, the substitution of surface-exposed and semi-exposed residues with alanine could provide a new and generic strategy for increasing a protein’s stability if we can determine the exact nature of the entropy destabilization and reduce its extent.

Finally, it is noteworthy that a protein in which more than 40% of the residues are alanines can fold into a native-like, well packed structure that can be crystallized and solved at high resolution. This observation implies that the determinants of a protein fold lie in residues deeply buried in the template structure. Indeed this study and our previous studies demonstrate that a substantial number of surface and semi-exposed residues do not actively contribute to specifying the native structure of proteins with densely packed interiors, which translates to highly cooperative thermal denaturation, a biophysical hallmark of natively folded proteins.

## Materials and Methods

### Protein expression and purification

All simplified BPTI variants were over-expressed using the pMMHA expression vector in *Escherichia coli JM109(DE3)pLysS* cell line, and collected by Ni-NTA chromatography. After removal of the Trp tag by CNBr cleavage, the BPTI variants were further purified by reverse phase HPLC as previously described^20^. Purified proteins were lyophilized and preserved at −30 °C until use. Protein identities were confirmed by ESI-TOF mass spectroscopy.

### Thermodynamic analysis

#### Sample preparation

Samples for circular dichroism (CD) and differential scanning calorimetry (DSC) were prepared by dissolving lyophilized proteins in 20 mM sodium acetate buffer (pH4.1, pH4.7, and pH5.5). All samples were filtered with a 0.20 μm membrane filter to remove aggregates that might have accumulated during dialysis. Protein concentrations and pHs were confirmed after dialysis and the samples were thoroughly degassed just before DSC measurements.

#### CD measurements

The CD measurements were performed at 20 μM protein concentration in 20 mM acetate buffer (pH4.7) at 4 °C, 40 °C and 70 °C using JASCO J-820 spectrophotometer. The reversibility of the thermal denaturation were assessed by measuring the CD at 222 nm wavelength while heating (forward) the samples to 80 °C, cooling (backward) them to 4 °C, and then re-heating the samples from 10 to 80 °C. All variants showed almost complete reversible thermal denaturation curves (Suppl. Figure S1).

#### DSC measurements

Samples at 1 mg/mL concentrations were dialyzed for 18 hours at 4 °C, as previously described^14^. DSC measurements were performed using a VP-DSC MicroCalorimeter (Microcal, MA, USA) at a scan rate of 1.0 °C/min in the temperature range of 5 to 90 °C. The individual apparent heat capacity curves were analyzed with a two-state model using a non-linear least-square fitting method and by assuming a linear temperature dependence of the heat capacity for the native and denatured states^21^,^22^,^23^ (Fig. 1).

### Structure analysis

#### Crystallization

Stock solution containing 10–15 mg/ml protein was prepared in 15 mM Tris-HCl, pH7.0. Crystals of all simplified BPTI variants were grown at 20 °C using the hanging drop vapor diffusion technique in 20–30% PEG4000, 0.2 M lithium sulfate and 0.1 M Tris-HCl (pH8.5).

#### Structure determination

The X-ray diffraction data were recorded from single crystals using a synchrotron beam line at the Photon Factory (KEK, Tsukuba, Japan). The data were processed with the HKL2000 program package, using DENZO for the integration and SCALEPACK for the merging and statistical analysis of the diffraction intensities^24^. The structures were determined by molecular replacement using 5PTI^13^ as a template with the program Molrep and refined using Refmac5^25^, as previously described^4^. Structures were validated using Molprobity^26^ and visualized using Coot^27^.

#### Identification of novel water molecules

The protein-protein and protein-water hydrogen bonds in the crystal structures were calculated using Molprobity^26^ and HBAT^28^. In short, hydrogen atoms were added to the x-ray structures using Molprobity and then hydrogen bonds were calculated using the HBAT program. Water molecules within 2 to 4 Å from the amide-nitrogen and carbonyl oxygen were considered to have strong protein-water hydrogen bonds (Figs 4 and 5), while water molecules within the 3.4 to 4 Å from Cβ-atoms were considered as methyl side-chain hydration (Fig. 5d). As a reference we also calculated the hydrogen bonds in 2SS-BPTI^29^ that contains SS-bonds at 5–55 and 14–38 sites, while the 30–51^th^ sites are substituted to alanines.

## Data Availability

The coordinates and structure factors of BPTI-21A, BPTI-22Ab, BPTI-23A and BPTI-24A variants are deposited in the Protein Data Bank under the PDB entry codes 4YPK, 4YPP, 4YR4 and 4YR5, respectively and we previously reported the structures of BPTI-19A (3AUB) and BPTI-20A (3CI7).

## Additional Information

**How to cite this article**: Islam, M. M. *et al*. Crystal structures of highly simplified BPTIs provide insights into hydration-driven increase of unfolding enthalpy. *Sci. Rep.* **7**, 41205; doi: 10.1038/srep41205 (2017).

**Publisher's note:** Springer Nature remains neutral with regard to jurisdictional claims in published maps and institutional affiliations.

## Supplementary Material

Supplemental Information

## Figures and Tables

**Table 1:** Thermodynamic parameters for reference BPTI and extensively simplified BPTI sequences.

Effect on thermodynamic parameters^1^					
Mutant ID	*T*_m_ (°C)	*∆T*_m_ (°C)	*∆H*_*T*m_ (kJ/mol)	*∆*H^vH^/*∆H*_cal_	*∆C_p_* (kJ/mol/K)
BPTI-[5,55] A14GA38V	52.73 ± 0.13	-	226.48 ± 0.69	0.98 ± 0.00	3.95 ± 0.24
BPTI-19A	51.80 ± 0.05	−0.93 ± 0.15	251.19 ± 0.41	0.99 ± 0.00	3.13 ± 0.16
BPTI-20A	48.07 ± 0.06	−4.66 ± 0.16	227.65 ± 0.38	1.00 ± 0.00	2.19 ± 0.16
BPTI-21A	45.06 ± 0.00	−7.67 ± 0.15	242.92 ± 0.02	0.98 ± 0.00	2.64 ± 0.00
BPTI-22Aa	42.83 ± 0.00	−9.90 ± 0.15	238.85 ± 0.03	0.98 ± 0.00	3.58 ± 0.01
BPTI-22Ab	47.86 ± 0.02	−4.87 ± 0.15	250.50 ± 0.17	0.99 ± 0.00	3.48 ± 0.08
BPTI-23A	45.85 ± 0.01	−6.88 ± 0.15	257.60 ± 0.33	0.98 ± 0.00	3.58 ± 0.29
BPTI-24A^2^	(44.50 ± NA)	(−8.23 ± 0.15)	(240.49 ± NA)	0.99 ± NA	(3.83 ± NA)
BPTI-25A^2^	(44.37 ± NA)	(−8.36 ± 0.15)	(240.34 ± NA)	0.99 ± NA	(3.85 ± NA)

^1^Thermodynamic parameters determined at pH4.7. *∆T*_m_ stands for differences in *T*_m_ from the reference stabilized BPTI-[5,55]A14GA38V. The thermodynamic parameters determined from DSC thermographs are mentioned. *∆C*_*p*_ values were determined from the slope of *T*_m_ versus *∆H*_*T*m_ plot.

^2^The DSC analysis of BPTI-24A and BPTI-25A were conducted only at pH4.7 and the thermodynamic parameters (including ***∆Cp)*** calculated from single DDCL analysis are shown. Values for BPTI-24 and 25 are estimates, not experimentally determined. ‘NA’ stands for ‘not available’. The reported errors are fitting errors computed by analyzing a single DSC curve (at pH4.7) using different initial *∆C*_*p*_ values.

**Table 2:** Effects of individual alanine substitution on specific thermodynamic parameters.

Mutant ID	Thermodynamic Parameters						
	Experimental (DSC)		Extrapolated at 37 °C^1^		Calculated (assuming a naïve additivity rule)^2^		
	*T*_m_ (K)	*∆H*_*T*m_(kJ/mol)	*∆H*_37 °C_ (kJ/mol)	*T∆S*_37 °C_ (kJ/deg.mol)	*T*m (K)	*∆H*_37 °C_ (kJ/mol)	*T∆S*_37 °C_ (kJ/deg.mol)
BPTI-[5,55] A14GA38V	325.88 ± 0.13	226.48 ± 0.69	164.67 ± 4.01	154.90 ± 3.87	-	-	-
BPTI-19A	324.95 ± 0.05	251.19 ± 0.41	204.80 ± 2.60	194.42 ± 2.50	-	-	-
BPTI-20A	321.22 ± 0.06	227.65 ± 0.38	203.45 ± 2.01	196.02 ± 1.94	321.22 ± 0.04	203.45 ± 2.18	196.02 ± 2.09
BPTI-21A	318.21 ± 0.00	242.92 ± 0.02	221.60 ± 0.05	215.71 ± 0.04	318.21 ± 0.06	221.60 ± 2.98	213.71 ± 2.86
BPTI-22Aa	315.98 ± 0.00	238.85 ± 0.03	217.96 ± 0.03	213.75 ± 0.03	315.98 ± 0.05	217.96 ± 3.02	213.75 ± 2.91
BPTI-22Ab	321.01 ± 0.02	250.50 ± 0.17	212.73 ± 0.90	204.90 ± 0.87	321.23 ± 0.05	229.57 ± 2.14	221.19 ± 2.05
BPTI-23A	319.00 ± 0.01	257.60 ± 0.33	225.93 ± 2.25	219.23 ± 2.22	319.00 ± 0.05	225.93 ± 2.14	219.23 ± 2.06
BPTI-24A^3^	(317.65 ± NA)	(240.49 ± NA)	(211.76 ± NA)	(206.43 ± NA)	(317.64 ± 0.06)	(211.76 ± 3.01)	(206.43 ± 2.89)
BPTI-25A^3^	(317.52 ± NA)	(240.34 ± NA)	(211.92 ± NA)	(206.67 ± NA)	(317.52 ± 0.06)	(211.92 ± 3.01)	(206.67 ± 2.89)

^1^Specific thermodynamic parameters at 37 °C calculated using *∆H*_*T*m_*, T*_m_ and *∆C*_p_** listed in Table 1.

^2^Effects of individual and/or pair alanine substitutions on the thermodynamic parameters at 37 °C calculated from the closest variant. For example, effects of the K15R17A substitution on thermodynamic parameters (*T*_m_, *∆H, T∆S* and *∆G*) were calculated as the difference between BPTI-19A and BPTI-19A-K15R17A (BPTI-21A). An alternate calculation based on a multi-linear equation is reported in Suppl. Table S1.

^3^The DSC analysis of BPTI-24A and BPTI-25A were conducted only at pH4.7 and the thermodynamic parameters (including ***∆C*p****) calculated from single DDCL analysis are shown (in parenthesis) and are estimates, not experimentally determined values. ‘NA’ stands for ‘not available’.

**Table 3:** Structure determination and refinement details.

Parameters	BPTI-19A*	BPTI-20A*	BPTI-21A	BPTI-22Ab	BPTI-23A	BPTI-24A
Space groups	C 121	P 2_1_2_1_2_1_	C 222_1_	C 222_1_	C 222_1_	C 222_1_
Matthews coefficient	2.10	2.14	2.16	2.18	2.27	2.42
Solvent content (%)	41.4	42.0	43.1	43.1	45.2	49.24
No. of reflection used	49180	38836	12300	23855	13898	14746
*R*-factor/*R*-free	0.16/0.17	0.15/0.21	0.16/0.21	0.14/0.18	0.15/0.21	0.17/0.23
Maximum resolution (Å)	1.00	1.39	1.99	1.59	1.90	1.89
Ramachandran plot statistics; Residues						
in the most favored region (%)	96.4	98.6	98.2	97.6	97.6	98.2
in outliers (%)	0.0	0.0	0.0	0.0	0.0	0.0
RMS deviation from wild-type BPTI at backbone atoms (Å)	0.430	0.429	0.336	0.312	0.308	0.304
RMS deviation among wild-type BPTIs at backbone atoms$ (Å)	0.359–0.396					

^*^We previously reported the structures of BPTI-19A (3AUB) and BPTI-20A (3Ci7.pdb).

^$^For wild-type BPTI, we used 4pti.pdb, 5pti.pdb, 6pti.pdb and 7pti.pdb.
