# Corrigendum: Crystal structures of highly simplified BPTIs provide insights into hydration-driven increase of unfolding enthalpy

**DOI:** 10.1038/srep46798

**Published:** 2017-05-09

**Authors:** Mohammad Monirul Islam, Masafumi Yohda, Shun-ichi Kidokoro, Yutaka Kuroda

Scientific Reports 7: Article number: 4120510.1038/srep41205; published online: 03 07 2017; updated: 05 09 2017

This Article contains errors in the Data Availability section, “The coordinates and structure factors of BPTI-21A, BPTI-22Ab, BPTI-23A and BPTI-24A variants are deposited in the Protein Data Bank under the PDB entry codes 4YPK, 4YPP, 4YR4 and 4YR5, respectively and we previously reported the structures of BPTI-19A (3AUB) and BPTI-20A (3CI7)”.

should read:

“The coordinates and structure factors of BPTI-21A, BPTI-22Ab, BPTI-23A and BPTI-24A variants are deposited in the Protein Data Bank under the PDB entry codes 5JB4, 5JB5, 5JB6 and 5JB7 respectively and we previously reported the structures of BPTI-19A (3AUB) and BPTI-20A (3CI7)”.

