# Supplementary material for: Crystal structures of highly simplified BPTIs provide insights into hydration-driven increase of unfolding enthalpy
Source: Sci Rep. 2017 Mar 7;7:41205. doi: 10.1038/srep41205 (PMC5339861; doi:10.1038/srep41205)
Supplement: Supplemental Information [file srep41205-s1.pdf]

Crystal structures of highly simplified BPTIs provide insights into hydration-driven increase of unfolding enthalpy

Mohammad Monirul Islam[#*], Masafumi Yohda[#], Shun-ichi Kidokoro[‡], Yutaka Kuroda[#$]

[#]Department of Biotechnology and Life Sciences, Tokyo University of Agriculture and Technology, 2-24-16 Nakamachi, Koganei-shi, Tokyo 184-8588, Japan; and [‡]Department of Bioengineering, Nagaoka University of Technology, Kamitomioka-cho, Nagaoka, Niigata 940-2188, Japan. [*]Department of Biochemistry and Molecular Biology, University of Chittagong, Chittagong-4331, Bangladesh.

[$]**Correspondence to Y.K.**
Email: ykuroda@cc.tuat.ac.jp
Phone/Fax: +81-42-388-7794

Figure Legends

Figure S1. Thermodynamic parameters of the simplified BPTIs. The Circular Dichroism (CD) spectra measurements at 20μM concentration in 20mM Acetate buffer, pH4.7 at 40°C (a) and at 70°C (b) are shown. (c) Mutant stabilities measured by CD. The CD measurements were done in 20mM acetate at protein concentration of 5-10μM at pH4.7. Symbols are shown within the panels.

Figure S2. Enthalpy-entropy compensation in multiple alanine substitutions. (a) Contribution of individual alanine substitution on changes in enthalpy ($\Delta\Delta H_{37°C}$ kJ/mol) and entropy ($\Delta T\Delta S_{37°C}$ kJ/mol) of protein unfloding are shown. (b) Additive effects of multiple alanine substitutions on enthalpy ($\Delta\Delta H_{37°C}$), entropy ($\Delta T\Delta S_{37°C}$) and Gibbs free energy ($\Delta\Delta GS_{37°C}$) where BPTI-[5,55] A14GA38V [12] is considered as the reference variant. For DSC analyses we used at least three data points for all BPTI variants, except the BPTI-24A and BPTI-25A for determining the experimental thermodynamic parameters (ΔH_{Tm}, *T*m *and* ΔC_p) (Table 1). The thermodynamic parameters were extrapolated at 37°C as described in Suppl. Information 2. Multiple alanine substitutions introduced unexpected enthalpy stabilization, which was however, over-compensated with entropy destabilization

Figure S3. Structural deviations in the simplified BPTI variants. (a) Pair wise RMS deviations of the backbone atoms from wild-type BPTI. Comparative view of main-chain temperature factors (b), side-chain temperature factors (c) and overall temperature factors (d) of wild-type and simplified BPTI variants are shown where color codes are as red: wild-type; green: BPTI-19A; blue: BPTI-20A; yellow: BPTI-21A; violet: BPTI-22Ab; cyan: BPTI-23A; and gray: BPTI-24A.

Figure S4. The conserved hydrogen bonds and hydration structures in the crystal structures of 2-SS BPTI and simplified BPTIs. Hydrogen bonds were claculated using HBAT (36). Arrows indicate the direction of hydrogen bonds, from donor to acceptor atoms. Resisues identities (as in crystal structures) and inter-atomic distances (Å) are mentioned. Despite multiple alanine substitutions, all the simplified BPTIs could retain the conserved hydrogen bonds and hydration structures present in wild-type BPTI structures.

Figure S5. Correlation between entropy destabilization ($T\Delta S_{37°C}$ kJ/mol) and methyl

side-chain hydration structures upon multiple alanine substitutions. The number of water molecules close (within 3.0-4.0 Å) to the side-chain Cβ-atoms were considered. As wild-type BPTI we considered 5pti, 6pti and 7pti structures and for the remaining variants we included all the chains (monomers) of their asymmetric unit (■ bars: $T\Delta S_{37°C}$ kJ/mol; ■ squares: number of water molecules close to Cβ atoms of Alanines; and □ squares: number of water molecules close to Cβ atoms of residues other than alanines).

Figure S1.

a)

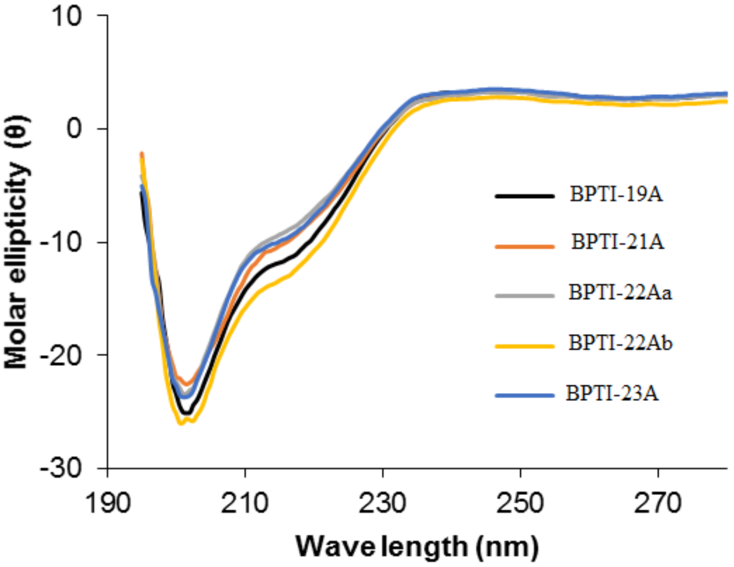

b)

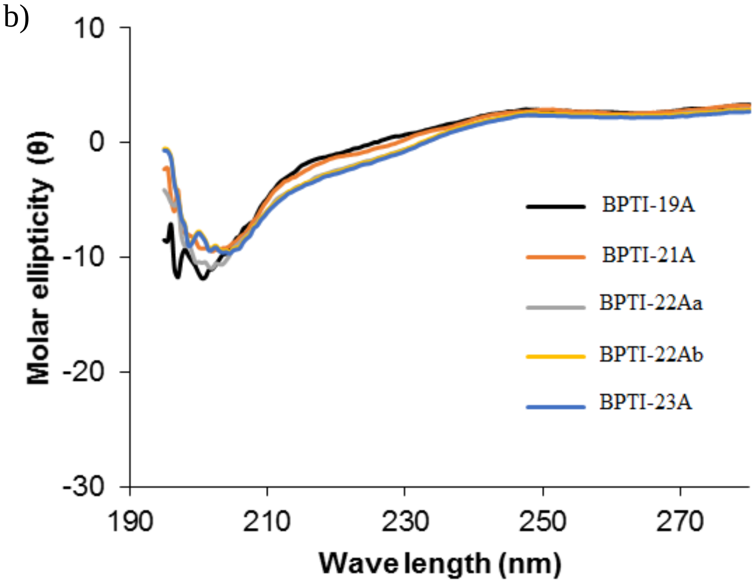

c)

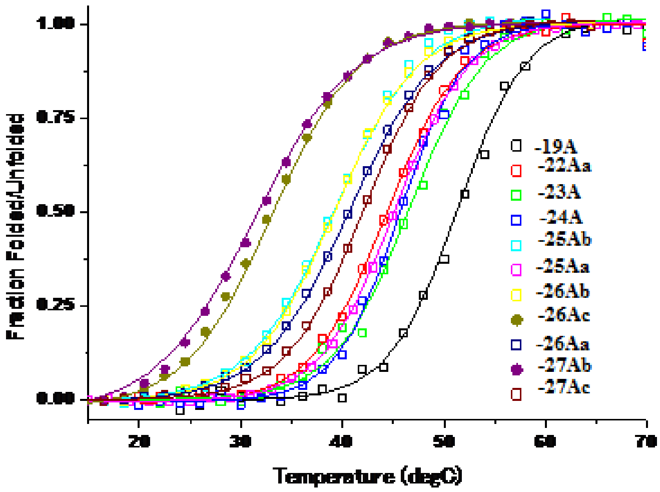

Figure S2

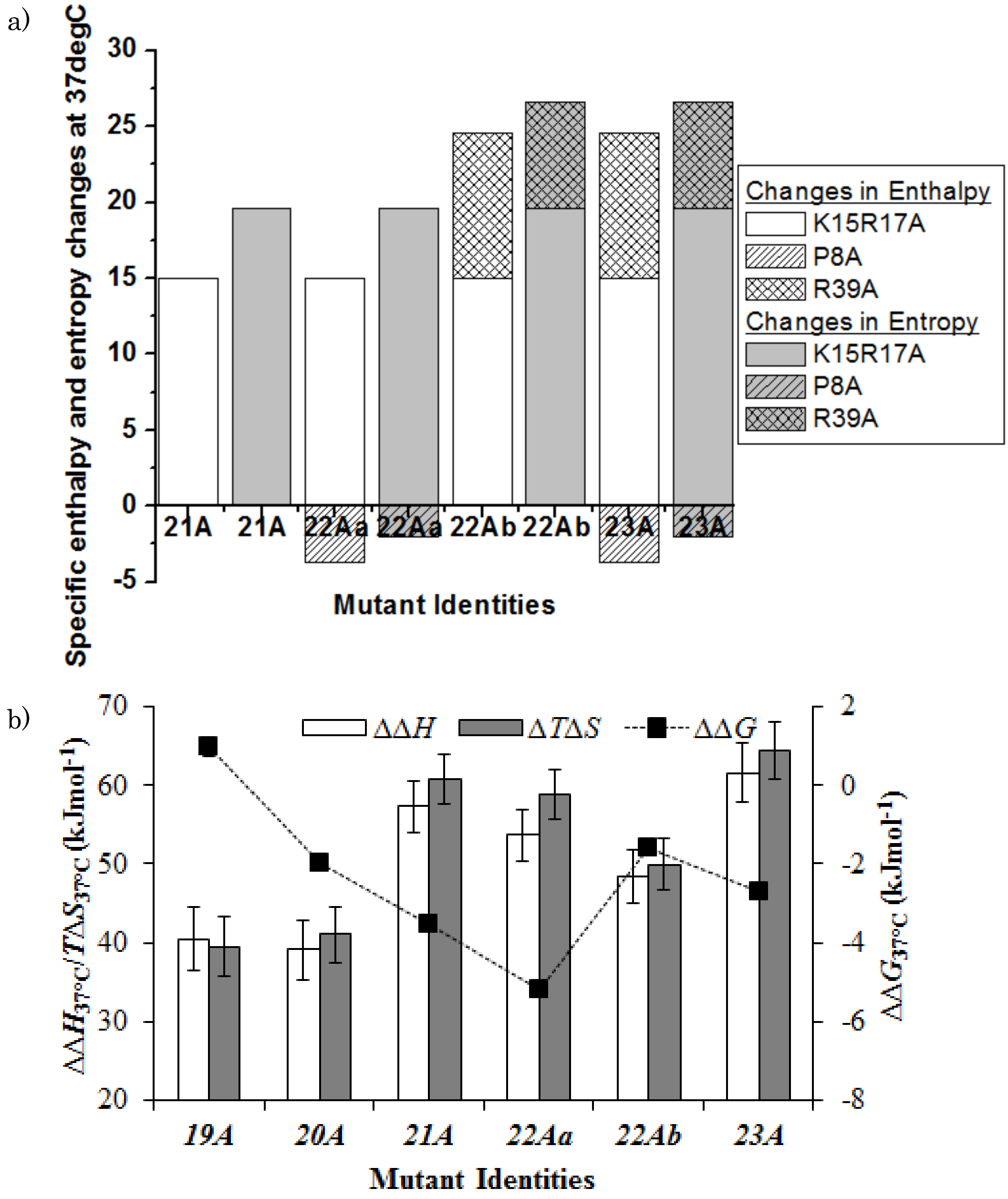
a)
Specific enthalpy and entropy changes at 37degC
Mutant Identities
21A
22Aa
22Ab
23A
Changes in Enthalpy
K15R17A
P8A
R39A
Changes in Entropy
K15R17A
P8A
R39A
b)
ΔΔH37°C/TΔS37°C (kJmol-1)
ΔΔG37°C (kJmol-1)
ΔΔH
ΔTΔS
ΔΔG
19A
20A
21A
22Aa
22Ab
23A
Mutant Identities

December 9, 2016

Figure S3.

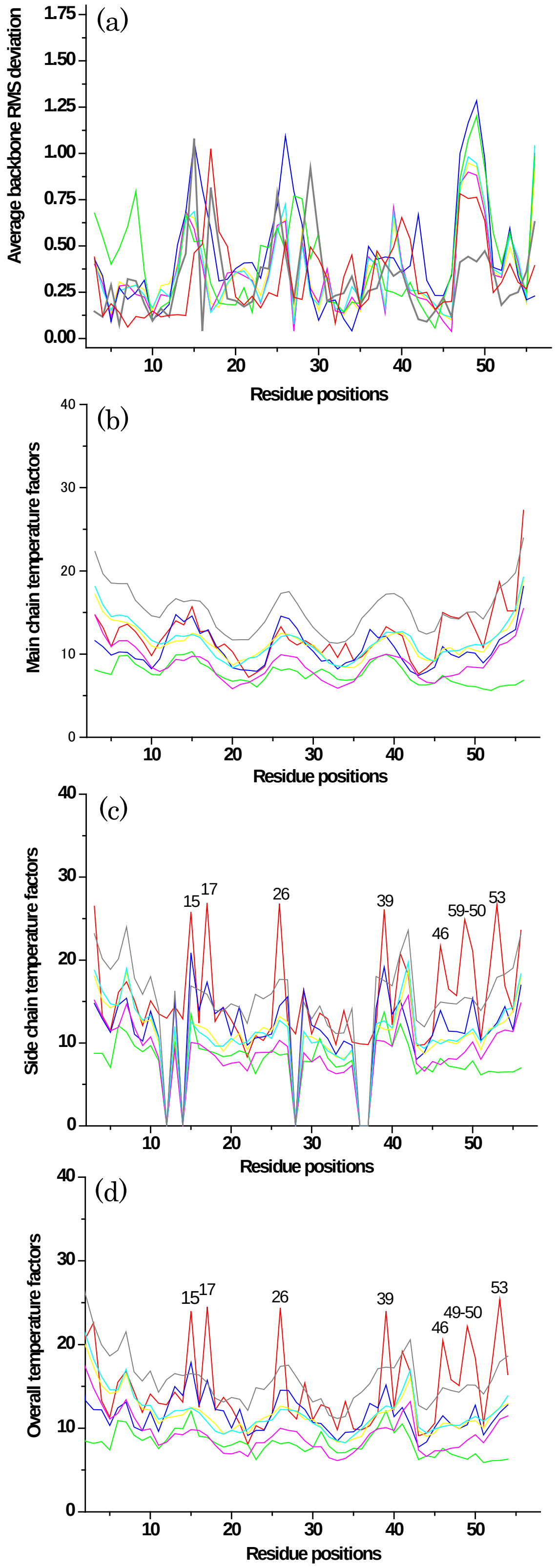

Figure S4

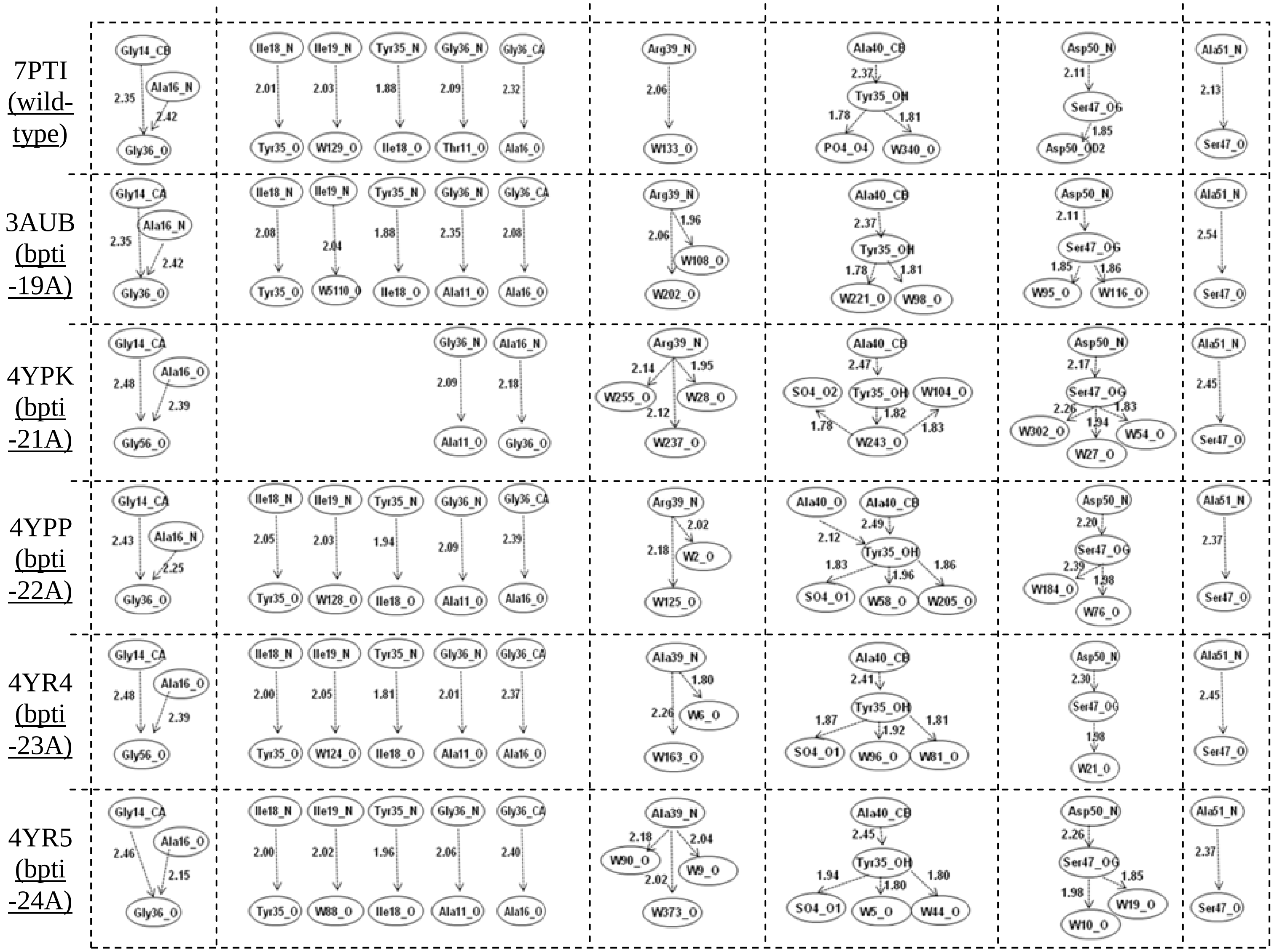
7PTI (wild-type)
3AUB (bpti -19A)
4YPK (bpti -21A)
4YPP (bpti -22A)
4YR4 (bpti -23A)
4YR5 (bpti -24A)

December 9, 2016

Figure S5

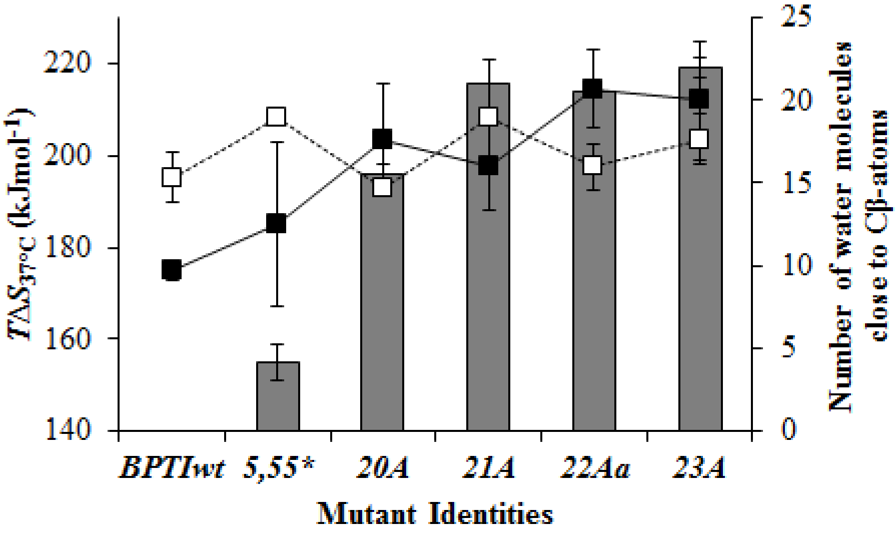

Table S1: Statistical Analysis of Thermodynamic Data.

Mutant Identities	Thermodynamic Parameters at 37°C						Melting Temperature (T_m °C)	
	$\Delta H_{37°C}$ (kJMol^{-1})		$T\Delta S_{37°C}$ (kJMol^{-1})		$\Delta G_{37°C}$ (kJMol^{-1})			
	Estimated$^{\$}$ (extrapolated)	Additivity Scheme$^{\#}$	Estimated$^{\$}$ (experimental)	Additivity Scheme$^{\#}$	Estimated$^{\$}$ (experimental)	Additivity Scheme$^{\#}$	Experimental	Additivity Scheme$^{\#}$
BPTI-19A	204.80±2.60	206.76±1.02	194.42±2.50	196.49±1.26	10.37±0.10	10.18±0.11	51.80±0.05	51.74±0.06
BPTI-20A	203.45±2.18	205.91±0.65	196.02±2.09	197.65±0.81	7.43±0.09	7.33±0.02	48.07±0.04	48.03±0.02
BPTI-21A	221.60±2.98	217.39±1.24	215.71±2.86	211.85±1.13	5.88±.012	5.84±0.03	45.06±0.06	45.04±0.01
BPTI-22Aa	217.96±3.02	215.09±1.31	213.75±2.91	210.43±1.22	4.22±0.12	4.23±0.01	42.83±0.05	42.82±0.01
BPTI-22Ab	229.57±2.14	222.44±0.43	221.19±2.05	215.82±0.17	8.37±.01	8.25±0.06	48.08±0.05	48.04±0.03
BPTI-23A	225.93±2.14	220.14±0.46	219.23±2.06	214.40±0.22	6.70±0.10	6.64±0.02	45.85±0.05	45.82±0.01
BPTI-24A$^{\$}$	(211.76±3.01)	(211.16±1.33)	(206.43±2.89)	(205.15±1.37)	(5.33±.12)	(5.31±0.01)	(44.50±0.06)	(44.48±0.01)
BPTI-25A$^{\$}$	(211.92±3.01)	(211.26±1.33)	(206.67±2.89)	(205.33±1.37)	(5.25±0.12)	(5.23±0.01)	(44.37±1.06)	(44.35±0.00)
Pearson correlation (r)	0.77*		0.82**		1.00***		1.00***	
Adjusted R Square (R^2)	0.53*		0.61**		0.99***		1.00***	
Regression coefficient (B value)	0.63*		0.72**		0.97***		0.99***	

$^{\$}$Thermodynamic values extrapolated to 37°C using original DSC data (ΔH_{Tm}, T_m and ΔC_p) determined from DSC thermographs. For DSC analyses we used at least three data points for all BPTI variants, except the BPTI-24A and BPTI-25A. For BPTI-24A and -25A, after analyzing the DSC curves at single data point (pH4.7), we observed that the ΔCp values were very much in line with increasing number on alanines in their sequences (Table 1). We thus decided to use these ΔCp values observed in single DDCL analysis for the calculation of specific thermodynamic

parameters at 37°C and estimated the thermodynamic parameters of BPTI-20A to -25A simple additivity of multiple alanine substitution; #predicted thermodynamic parameters obtained from regression analysis of estimated thermodynamic parameters (aditivity scheme) and experimental parameters (extrapolated values). First, the differences observed in thermodynamic parameters due to different alanine substitution were assigned to the specific alanine substitution and then the effects of individual alanine substitutions are combined in a simple additive fashion to predict the thermodynamic parameters of multiple alanine substitutions.

Specifically, we first estimated the contribution of individual alanine substitutions (BPTI-21A to -25A) on enthalpy, entropy, free energy and melting temperature (see Table 2) considering BPTI-19A as a template. Then, the contribution of individual residues on thermodynamic parameters was added to those of BPTI-19A in accordance with the sequences of BPTI-21A to -25A to investigate the additive nature of thermodynamic parameters upon multiple alanine substitutions. We used a simple additivity scheme (as suggested by a reviewer, $H=H0+h1*n1+h2*n2+h3*n3+h4*n4+h5*n5$, where $h(i)$ is the thermodynamic contribution of a given Ala substitution and $H0$ is the thermodynamic parameter of the template BPTI-19A). Melting temperatures at pH4.7 from DSC thermograms are shown. Statistical analyses were carried out using SPSS v23.0 and Pearson correlation (r), Adjusted R square (R^2) and regression coefficient (B values) are shown. ***, **, and * stand for p=<0.001, p=<0.01 and p=<0.05 respectively. The low adjusted R-square may report some over-fitting since six fitting parameters were used to fit eight data points. All thermodynamic parameters ($\Delta H_{37°C}$, $T\Delta S_{37°C}$, and $\Delta G_{37°C}$) calculated using standard additivity scheme are almost the same to those estimated at 37°C using original thermodynamic parameters (ΔH_{Tm}, T_m and ΔC_p), clearly indicating the additivity of multiple alanine substitutions. $The DSC analysis of BPTI-24A and BPTI-25A were conducted only at pH4.7 and the thermodynamic parameters (including ΔC_p) calculated from single DDCL analysis are shown (in parenthesis), values shown are estimates, not experimentally determined
